# The role of individual, household, and area of residence factors on self-rated health in Colombian adults: A multilevel study

**DOI:** 10.7705/biomedica.4818

**Published:** 2020-06-30

**Authors:** Beatriz Caicedo-Velásquez, María Clara Restrepo-Méndez

**Affiliations:** 1 Facultad Nacional de Salud Pública, Universidad de Antioquia, Medellin, Colombia Universidad de Antioquia Facultad Nacional de Salud Pública Universidad de Antioquia Medellin Colombia; 2 Grupo de Investigación Epidemiología y Bioestadística, Universidad CES, Medellin, Colombia Universidad CES Grupo de Investigación Epidemiología y Bioestadística Universidad CES Medellin Colombia; 3 Oxford Maternal and Perinatal Health Institute (OMPHI), University of Oxford, Oxford, England Oxford Maternal and Perinatal Health Institute (OMPHI) University of Oxford Oxford England

**Keywords:** Residence characteristics, social conditions, multilevel analysis, adult, Colombia., características residenciales, condiciones sociales, análisis multinivel, adulto, Colombia.

## Abstract

**Introduction:**

Self-rated health is strongly associated with morbidity and mortality. It is largely influenced by individual factors but also by individuals’ social surroundings and environment.

**Objective:**

To investigate individual, household, and locality factors associated with self- rated health in Colombian adults.

**Materials and methods:**

We conducted a cross-sectional multilevel study using data from national databases on 19 urban localities and 37,352 individuals nested within 15,788 households using a population-based survey. Given the natural hierarchical structure of the data, the estimates of self-rated health related to individual, household, and locality characteristics were obtained by fitting a three-level logistic regression.

**Results:**

The adjusted multilevel logistic models showed that at individual level, higher odds of poor self-rated health were found among older adults, persons from low socio-economic status, those living without a partner, with no regular physical activity, and reporting morbidities. At the household level, poor self-rated health was associated with households of low socioeconomic status located near noise sources and factories and in polluted and insecure areas. At the locality level, only poverty was associated with poor self-rated health after adjusting for individual and household variables.

**Conclusions:**

These results highlight the need for a more integrated framework when designing and implementing strategies and programs that aim to improve health conditions in urban populations in Latin America.

## Resumen

**Introducción.:**

La autopercepción de la salud se asocia con la morbilidad y la mortalidad debido principalmente al efecto de las condiciones individuales y las características sociales y del ambiente en el que viven las personas.

**Objetivo.:**

Investigar los factores individuales, del hogar y de la localidad asociados con la autopercepción de la salud en adultos colombianos.

**Materiales y métodos.:**

Se llevó a cabo un estudio transversal. La información sobre las 19 localidades urbanas consideradas se obtuvo de bases de datos nacionales, en tanto que los datos sobre los 37.352 individuos anidados en 15.788 hogares provinieron de una encuesta de base poblacional. Dada la estructura jerárquica de los datos, las estimaciones del efecto de las variables individuales, del hogar y de la localidad sobre la autopercepción de la salud se hicieron utilizando un modelo de regresión logística de tres niveles.

**Resultados.:**

Los modelos multinivel ajustados evidenciaron que a nivel individual había una mayor probabilidad de tener una peor percepción de la salud entre adultos mayores, personas de bajo nivel socioeconómico, sin compañero, físicamente inactivos y con enfermedades. A nivel de hogar, la peor percepción de la salud se asoció con la pertenencia a familias de bajo nivel socioeconómico, residentes cerca de fábricas, áreas contaminadas, inseguras y de alto ruido. Por último, a nivel de localidad y después del ajuste por variables individuales y del hogar, la residencia en localidades pobres aumentó la probabilidad de tener una peor percepción de la propia salud.

**Conclusiones.:**

Los resultados evidencian la necesidad de considerar un marco conceptual más amplio en el momento de diseñar e implementar estrategias y programas que apunten al mejoramiento de las condiciones de salud de las poblaciones urbanas en Latinoamérica.

Self-rated health is an indicator of the quality of life that Is related to the self-perception of an individual's health status ^1^^,^^2^. Self-perception Is a practical method for collecting information on individual health since it entails a single aspect: a subject’s perception of his or her health status ^1^. This information is useful for planning, implementing, and monitoring health initiatives and programs as it has been found that self-rated health is strongly associated with morbidity and mortality ^3^^-^^8^, a relationship that remains after adjusting for physical, sociodemographic, and behavioural factors ^3^^-^^8^.

Self-rated health is influenced by individual factors such as sex, age, race, ethnicity, education levels, wealth, and employment status ^1^^,^^9^^-^^13^. Furthermore, common factors among groups of people like their social surroundings and environment play a role in the self-perception of their health ^14^^-^^23^.

It should be mentioned that the term contextual or neighbourhood effects involves both structural and social aspects. Structural characteristics refer to the physical and natural environment where individuals live measured through socio-demographic characteristics such as poverty, family structure, unemployment, and the availability of education, employment, transportation, health care, grocery shopping, and recreational services ^24^. The social characteristics refer to the social and organizational processes or collective aspects of community life that may influence residents’ behaviour ^19^, in other words, social networks, control, cohesion, norms for support, perceptions of violence, and collective efficacy ^25^. Some studies that have examined the influence of neighbourhood-level factors on self-rated health indicate that deprivation, lower socio-economic status, poor-quality residential environment and transport, drug misuse, rubbish on the streets, unsafety, and dissatisfaction with green spaces are associated with fair to poor self-rated health ^3^^,^^17^^,^^26^^-^^30^.

The mechanisms of the relation between contextual factors and poor self-rated health are not clear. Some authors suggest that neighbourhood problems may constitute a source of chronic stress, which may increase the risk of poor self-perception of health ^3^^,^^31^. Documenting contextual factors that may contribute to modify such perception is important for the design and implementation of effective prevention strategies and interventions. It is important to better understand how group and individual factors interrelate in predicting self-rated individual health. In general, researchers in this field have not properly taken into account the role of individual and family influences while those focused on individual and family influences have generally disregarded the role of neighbourhood influences. Therefore, we need to develop conceptual frameworks incorporating various levels of analysis, as well as due consideration to potential mechanisms of relation as the lack of an appropriate theoretical framework may lead to deceptive conclusions. A common approach to such analysis is the multilevel design and its hierarchical model that allows the integration of independent variables from different levels of analysis ^32^^-^^35^.

There are relatively few studies in Latin America using an approach that simultaneously considers how individual and contextual aspects contribute to self-rated health status ^1^^,^^16^^,^^36^ and even more scarce in Colombia, especially in Bogotá, which is considered one of Latin America’s largest metropolitan areas. Understanding these associations is relevant in the light of the current Colombian ten-year public health plan and the Sustainable Development Goals for a healthy life and well-being for all (SDG 3), and inclusive, safe, resilient, and sustainable cities and human settlements (SDG 11). Consequently, this study aimed at examining the relationships between poor self-rated health and individual, family, and locality factors among Colombian adults living in Bogotá especially focused on the effects of locality structural and social conditions based on a conceptual model linking locality characteristics and poor self-rated health and on a multilevel model to evaluate the effect of locality conditions in adults’ poor self-rated health and the interaction between individual and family factors. To our knowledge, this is the first study that explores contextual effects and self-rated health among adults in a Colombian urban context.

## Materials and methods

### Design and study population

Bogotá is the capital city of Colombia with a population of 7,467,000 inhabitants of whom 99% live in the urban area. The city is divided geographically and administratively into 20 localities. For the analysis, we used data from the multipurpose survey carried out in Bogotá in 2011 by the Departamento Administrativo Nacional de Estadística, DANE ^37^. The survey is a population-based study that collects information on the social, economic, and general living conditions of the population in Bogotá’s 19 localities (excluding the semi-rural area of Sumapaz).

We used a probabilistic clustered sample stratified by socioeconomic status where the observational units were the households and non-institutionalized individuals. The parameters for sample estimation were 5% relative standard error, 95% confidence level, and 10% prevalence of the main health indicators. The detailed account of the methods used for this population survey is available ^37^^,^^38^. We used a cross-sectional design including 20-year-old and older individuals who completed the interview and provided information on their self-rated health. These data respond to a natural hierarchy structure with 37,352 individuals nested within 15,788 households from 19 urban localities.

### Study variables

We used a questionnaire to collect a wide range of demographic and socio-economic information about individual and household conditions. Self-rated health was classified on a 1 to 4 scale: ‘Very good’, ‘good’, ‘poor’, and ‘very poor’. Previous studies have suggested that self-rated health is a reliable indicator of an individual’s current health with high predictive validity ^39^. To compare the results with those from other studies the original categories were recorded into a binary outcome: 0 for very good and good, and 1 for poor or very poor.

The following individual characteristics were also taken from the questionnaire and included in the analysis as independent variables: gender (male, female), age (20-29, 30-39, 40-49, 50-59, >60), schooling (<5, 6-11, >11 years), marital status (with or without a partner), working mainly in the last week (yes/no), regular physical activity (yes/no), and morbidities (none, one- two, or three or more of the following morbidities: cardiovascular diseases, respiratory diseases, kidney diseases, digestive diseases, arthritis, diabetes, malign tumours, mental diseases, or asthma/allergies). At the household level, we explored the effect of socio-economic status (low, middle, high), living in a noisy area (yes/no), contamination problems in the area (yes/no), insecurity (yes/no), close to rubbish dumps (yes/no), close to factories (yes/no), and presence of illicit drug markets (yes/no).

Data on the socio-economic characteristics of the locality were taken from official national datasets reported by DANE ^37^^,^^40^. At the locality level, we included:

1) quartiles of the Gini coefficient to measure the level of income inequality in the localities (values between 0 and 1) where the lowest quintile represents more equal localities and the highest one more unequal localities;

2) quartiles of poverty measuring the proportion of residents with disadvantageous life conditions regarding schooling, employment, access to health services, and housing where localities were categorized into quintiles, with the lowest quintile comprising the richest group of localities and the highest one the poorest localities ^40^;

3) homicide rate (per 100,000 inhabitants);

4) the percentage of population perceiving increased insecurity in their locality, and

5) population density defined as the number of residents per square meter (m^2^).

### Conceptual model

The analysis was based on a hierarchical conceptual model that not only considered a proposed hierarchy of causal relationships but also used criteria for selecting variables beyond purely statistical considerations ^41^. At the individual level, the most distal determinants were age and gender, schooling, marital status, and work-related status. The second level included the effects of being physically active and morbidities. At the household level, we included the effects of socio-economic status and location characteristics (living close to a noisy area with pollution problems, insecurity, and exposure to rubbish dumps, close to factories, and to illicit drug markets. Finally, at the locality level, we examined the distal effect of social conditions (Gini coefficient and poverty) which in their turn may have had a direct influence on the variables at the second level, the effects of the homicide rate, percentage of population perceiving an increase in insecurity in their locality, and population density.

Analytical procedures: Hierarchical analysis with a multilevel logistic model.

Estimates of poor self-rated health associated with individual, household, and locality characteristics and their respective 95% confidence intervals were obtained by fitting a logistic regression model with random intercept, fixed coefficients, and a three-level structure with individuals at level 1, households at level 2, and localities at level 3:



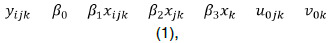



where is the poor self-rated health condition for individual *i* in household *j* within locality *k*. The log-probability of poor self-rated health for all individuals in all localities is represented by . The individual, household, and locality variables are represented by , and their regression coefficients by , respectively. These were transformed into odds ratios to facilitate comparisons. Finally, the household- and locality-level random effects are represented by and and they measure household and locality differences conditioned to the variables that are specified in the model. We assumed a normal distribution with the respective variances: expressed on a logit scale.

The level 1 unexplained variance, , assumes a Bernoulli distribution given the binary nature of the response. To indicate the percentage of variance due to differences among localities, the intra-class correlation coefficient was estimated using the ratio of the locality-level variance and the total variance. Moreover, to better quantify the effects of locality and understand their size, the locality-level variance was transformed to a median odds ratio (MOR) ^42^ by translating the locality-level variance into an odds ratio that quantified the variation among localities randomly choosing and comparing any two individuals from two different localities. This may be interpreted as the increased risk of poor self-rated health that an individual would have on average if he or she moved to another locality with a higher risk of poor health ^43^, which was estimated as:



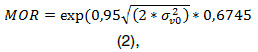



where 0.6745 is the 75^th^ percentile of the cumulative distribution function of the standard normal distribution. The uncertainty interval of the MOR (Bayesian confidence intervals) was derived from the monitoring chain of the MCMC estimates and the above equations ^44^.

In the hierarchical conceptual model, the analysis consisted of a sequence of six models of growing complexity. The first model was a null model or model without covariates. The second one included the effect of the most distal determinants at individual level (age and gender, schooling, marital status, and work-related status). The third model included the effects of being physically active and morbidities. The fourth one explored the effect of household conditions (socio-economic status, location, and characteristics). Finally, in the last model, we examined the effect of the Gini coefficient and poverty, as well as the effect of the percentage of population perceiving an increase in insecurity of the locality and population density.

We used chi-squared tests with a significance level of 20% to identify potential confounders. This pre-specified cut-off value has proven to better identify the presence of confounding effects than using a cut-off of 0.05 ^45^^,^^46^. We also used a chi-squared test for heterogeneity to analyze nominal variables. For ordinal variables, such as the neighbourhood variables, for which we hypothesized dose-response effects, we used a linear trend test.

Given that the urban localities to characterize differences and estimate the effect of some associated factors were only 19, models were estimated with full Bayesian procedures to estimate exactly the parameters ^44^.

To validate the results of the random-effects model, the multilevel logistic model was re-specified as a fixed-effects model and the DIC index was used to choose between the fixed and random effects approach where the model with the lower DIC was preferred as a trade-off between complexity and fitness ^47^. Both methods gave similar point estimates, but the DIC index indicated better performance for the multilevel method (data not shown). The results we are presenting corresponded to the multilevel regression analysis.

The analyses were done with the MLwiN v2.31 statistical software package using the runmlwin command ^48^ with full Bayesian MCMC methods and minimally informative priors. Following Draper’s ^49^ good-practice recommendations, a burn-in of 500 iterations was used with monitoring for another 50,000 iterations.

The analyses were based on publicly available data from a national survey and official national datasets. Ethics procedures were the responsibility of the institutions that commissioned, funded, or administered the surveys/data collection.

## Results

Table 1 describes individuals, households, and locality characteristics. More than half of the respondents were women, 48% were aged 20 to 39 years old, a third had more than 11 years of schooling, two thirds had a partner, and 67% reported having a job during the previous week. Regarding their health conditions, around a fifth of the individuals reported being physically active and 60% reported not suffering from morbidities. Most of the households were located in middle- and low-socioeconomic status areas. A third of them were located in areas with noise, contamination, and insecurity problems, close to rubbish dumps, industries, and illicit drug markets. On average, localities had a population density of 181 inhabitants/m^2^, a homicide rate of 42 homicides per 100,000 inhabitants, 45% of their residents perceived an increase in insecurity, the Gini coefficient at the lowest inequality quartile was 0.39 while at the highest it was 0.55. At least 22% of the population in the poorer localities lived in poverty.

**Table 1.** Studied population according to individual, household, and locality characteristics, Bogota, Colombia, 2011

Variable	n	%
Individual level (n=37,352)		
Gender		
Male	16,846	45.10
Female	20,506	54.90
Age		
20-29	9,681	25.92
30-39	8,155	21.83
40-49	7,475	20.01
50-59	5,824	15.59
≥60	6,217	16.64
Schooling (years with passing scores)		
<=5	10,575	29.13
6-11	13,644	37.58
> 11	12,088	33.29
Marital status		
With a partner	24,53	65.67
Without a partner	12,822	34.33
Working		
Yes	25,127	67.27
No	12,225	32.73
Regular physical activity		
Yes	7,939	21.25
No	29,413	78.75
Morbidities		
0	22,48	60.18
1-2	12,735	34.09
>=3	2,137	5.72
Household level (n=15,788)		
Socio-economic status		
Low	6,829	43.30
Middle	7,974	50.50
High	780	4.90
Located within a noisy area		
Yes	6,269	39.70
No	9,515	60.30
Located within an area with contamination problems		
Yes	7,517	47.60
No	8,269	52.40
Located within an area with insecurity problems		
Yes	12,095	76.60
No	3,696	23.40
Close to rubbish dump		
Yes	2,047	13.00
No	13,741	87.00
Close to factories		
Yes	3,426	21.70
No	12,362	78.30
Close to drug markets		
Yes	4,403	27.90
No	11,385	72.10
Locality level (n=19)		
Gini coefficient		
Quartile 1	5	0.39
Quartile 2	4	0.42
Quartile 3	5	0.48
Quartile 4	5	0.55
Population in poverty (%)		
Quartile 1	6	5.61
Quartile 2	5	10.30
Quartile 3	4	16.00
Quartile 4	4	21.90
Mean (SD) homicide rate x 100,000	42.35	(36.35)
Mean (SD) of population perceiving an increase in the insecurity (%)	45.05	(7.65)
Mean (SD) population density (m^2^)	180.72	(56.41)

SD: Standard deviation

m^2^: Squared meter

**Figure 1:**
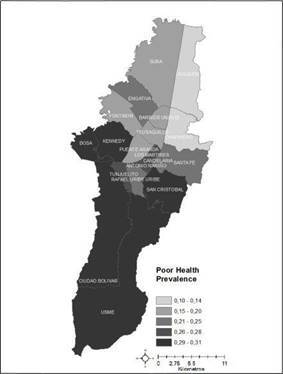
Predicted prevalence of poor health by locality of residence, Bogota-Colombia, 2011

The prevalence of poor self-rated health in the population was 24% (95% confidence intervals: 21%-26%). We found strong evidence of variation on the prevalence of poor self-rated health among localities (pcO.001). Figure 1 shows the scale of the differences plotting the distribution of localities according to the intervals of the prevalence of poor self-rated health predicted from the null model by means of the simulation-based procedures of the MLwiN customized predictions ^50^. The differential prevalence showed that individuals in northern Bogota had better self-rated health than those from southern Bogota.

The locality-level variance of 0.09 translated into a 1.31 MOR (95% Cl: 1.16-1.42), which suggests significant differences between localities. For instance, if an individual were to move from a locality with a low prevalence of poor self-rated health to one with high prevalence, his or her individual odds were around 31% greater than if he or she stayed in a lower risk locality.

Table 2 shows the prevalence of poor self-rated health by independent variables. The crude analysis showed that individuals older than 60 years had around 65% higher odds of having poor self-rated health compared to those aged 20-29 years. Having less than five years of education increased five times the odds of poor self-rated health compared to those with 11 years or more. Similarly, women, people without a partner and/or a job during the previous week, those physically inactive, and those suffering from three or more morbidities had much higher odds for poor self-rated health when compared to their reference categories. Additionally, people living in households from low socio-economic status noisy areas, with contamination and insecurity problems, close to rubbish dumps, factories, and illicit drug markets showed greater odds for poor self-rated health when compared with their reference categories. Similarly, localities with low inequality, high poverty level, high rate of homicides, and high population density showed greater odds for poor self-rated health. The insecurity level of the localities was not associated with self-rated health.

**Table 2:** Prevalence, crude, and adjusted analyses of the association between individual, household, and locality-related variables and poor or very poor health, Bogota D.C., Colombia, 2011

Crude analysis				Adjusted analysis**	
Variable	Prevalence	OR (CI 95%)	p-value	OR (CI 95%)	p-value
Individual level					
Gender			0.002		0.2
Male	24.05	Reference		Reference	
Female	26.43	1.08 (1.03; 1.14)		1.03 (0.98; 1.09)	
Age			<0.001		<0.001*
20-29	22.21	Reference		Reference	
30-39	18.12	0.85 (0.78; 0.93)		0.78 (0.72; 0.85)	
40-49	22.96	1.04 (0.96; 1.13)		1.05 (0.97; 1.13)	
50-59	29.38	1.28 (1.18; 1.40)		1.33 (1.23; 1.44)	
≥60	38.88	1.65 (1.52; 1.80)		1.46 (1.35; 1.59)	
Schooling (years with passing scores)			<0.001		<0.001*
<=5	43.35	4.89 (4.38; 5.45)		3.75 (3.49; 4.03)	
6-11	23.36	1.95 (1.76; 2.16)		1.87 (1.74; 2.01)	
> 11	13.33	Reference		Reference	
Marital status			<0.001		<0.001
With a partner	22.48	Reference		Reference	
Without a partner	30.87	1.62 (1.49; 1.75)		1.27 (1.20; 1.34)	
Working			<0.001		<0.001
Yes	17.99	Reference		Reference	
No	40.51	3.19 (2.94; 3.46)		2.38 (2.25; 2.51)	
Regular physical activity			<0.001		<0.001
Yes	21.15	Reference		Reference	
No	26.5	1.25 (1.14; 1.37)		1.41 (1.32; 1.51)	
Morbidities			<0.001		<0.001*
0	11.46	Reference		Reference	
1-2	41.85	5.65 (5.16; 6.19)		5.00 (4.72; 5.31)	
>=3	73.33	22.93 (19.11; 27.51)		17.02 (15.17;19.08	
Household level					
Socio-economic status			<0.001		<0.001*
Low	30.03	2.90 (2.28; 3.70)		2.21 (1.78; 2.74)	
Middle	22.67	2.05 (1.62; 2.59)		1.75 (1.42; 2.14)	
High	8.71	Reference		Reference	
Located within a noisy area			<0.001		<0.001
Yes	28.53	1.32 (1.22; 1.43)		1.12 (1.06; 1.19)	
No	23.27	Reference		Reference	
Located within an area with contamination problems			<0.001		<0.001
Yes	27.95	1.29 (1.20; 1.40)		1.15 (1.08; 1.22)	
No	23.00	Reference		Reference	
Located within an area with insecurity problems			<0.001		<0.001
Yes	27.47	1.53 (1.39; 1.69)		1.20 (1.12; 1.30)	
No	17.98	Reference		Reference	
Close to rubbish dumps			<0.001		0,889
Yes	31.57	1.31 (1.17; 1.47)		1.01 (0.93; 1.09)	
No	24.42	Reference		Reference	
Close to factories			0.01		<0,001
Yes	27.81	1.53 (1.39; 1.69)		Reference	
No	24.65	Reference		Reference	
Close to drug markets			<0.001		<0,001
Yes	30.87	1.53 (1.39; 1.69)		1.18 (1.11; 1.26)	
No	23.24	Reference		Reference	
Locality level					
Gini coefficient			0.004		0,588*
Quartile 1	29.72	1.73 (1.26; 2.39)		1.27 (0.97; 1.65)	
Quartile 2	25.05	1.36 (0.96; 1.91)		1.08 (0.93; 1.27)	
Quartile 3	25.33	1.39 (1.00; 1.91)		1.09 (0.94; 1.28)	
Quartile 4	19.79	Reference		Reference	
Population in poverty (%)			<0.001		0.003*
Quartile 1	18.84	Reference		Reference	
Quartile 2	25.54	1.50(1.18; 1.92)		1.31 (1.12; 1.52)	
Quartile 3	27.64	1.68 (1.30; 2.18)		1.25 (1.06; 1.47)	
Quartile 4	30.61	1.96 (1.51; 2.53)		1.36 (1.15; 1.61)	
Mean (SD) homicide rate x 100,000		1.00 (1.00; 1.01)	0.04	1.00 (0.99; 1.00)	0.476
Mean (SD) of population perceiving an increase in the insecurity (%)		1.01 (0.98; 1.02)	0.57	0.99 (0.97; 1.01)	0.771
Mean (SD) population density (m^2^)		1.00 (1.00; 1.01)	0.01	0.99 (0.96; 1.00)	0.570

* Wald test for linear trend

** Adjusted for all variables

in the same level or in

higher levels with p<0.2

SD: Standard deviation

CI: Confidence interval

Adjusted analyses were carried out according to the hierarchical levels described in the methods section. After adjustment, the odds of poor self- rated health for people older than 50 years remained higher when compared to those aged 20-19 years old. The association with the years of education, not having a partner and/or a job, being physically inactive, and suffering from three or more morbidities also remained significant. The association of residing in poor households in areas with noise, contamination, and security problems, close to factories and illicit drug markets was virtually unaltered by adjustment. We found a dose-response relationship between locality poverty and odds of poor self-rated health. Otherwise, the association of gender, household close to rubbish dumps, Gini coefficient, homicide, and population density with poor self-rated health disappeared after adjustment (table 2).

In the adjusted model, the between-locality variance decreased to 0.004. This equates to a 1.06 MOR (95% Cl: 1.00; 1.11), which means that after considering the effects of individual, household, and locality characteristics there are no unexplained differences between localities. This result is also shown by the adjusted ICC of 0.048% in the final model.

## Discussion

We examined the effect of context- and individual-related variables on poor self-rated health in Colombian adults living in a metropolitan area. Our results confirmed that the characteristics of individuals, households, and place of residence influenced individual health perception.

We found greater odds of poor self-rated health in individuals aged 50 years or older, those with lower education, without a partner and/or employment, non-physically-active individuals on a regular basis, and those who reported more than three morbidities. After adjusting for individual characteristics, household characteristics were also associated with poor self-rated health. Low socioeconomic households located in areas with problems of noise, pollution, neighbourhood insecurity, and near factories or illicit drug markets showed greater odds of poor self-rated health. Additionally, localities with a higher proportion of poverty showed greater odds of poor self-rated health independently from individual and household factors. These findings are consistent with other studies showing strong associations between physical conditions of the place of residence and individual health ^3^^,^^16^^,^^17^^,^^21^^,^^32^^,^^51^. In general, poorer areas usually present characteristics that are unfavourable to good health such as Inadequate healthcare networks, absence of areas for practicing physical activities, a poorly organized physical environment (accumulated garbage, dirtiness, pollution, noise, overcrowding), deficient basic sanitation, transportation, and education, insufficient levels of social cohesion and participation, and greater exposure to violence ^16^^-^^18^^,^^23^^,^^32^^,^^51^.

The main methodological limitation of studies that investigate context- related characteristics is the definition of the geographic area whose characteristics may be relevant to the specific health outcome under study ^18^. As we were interested in analyzing the association of the physical environment and the structural characteristics of the place of residence with self-perceived health, the geographically administrative definition of localities was relevant to us as we think it validates the individual perception of these areas to a certain extent given that the localities are previously defined political and administrative units and, therefore, a natural grouping for the respondents. The greatest advantage of using this geographical unit in the analysis is the feasibility of obtaining variables measured at that level. The main disadvantage is that such grouping may not reflect the true context in which individuals are exposed to contextual risks. An additional limitation concerns the study design. Studies with a cross-sectional design are limited to identifying associations rather than causal relationships and reverse causality may exist, especially at individual-level variables. For instance, it can be argued that individuals reported poorer health because they were unemployed or that they were unemployed because of their poor health. Thus, longitudinal studies are important to confirm the associations reported here. Furthermore, we cannot rule out the possibility of residual confounding factors that were not explored, such as individual income or wealth, and of lack of precision in certain measurements of socioeconomic characteristics, such as education and employment status.

Stratified analyses exploring sex differences may have been important in previous studies as they showed that self-reported health and the use of health services was worse in women than in men ^16^^,^^52^. Those findings are important as they suggest that women might benefit more from better health services or suffer more due to a lack of them in a wealthy or poor area, respectively. However, we did not find differences by sex in our results (data available on request). Additional analyses exploring conflicting findings among studies regarding sex differences are needed.

To understand the multifaceted nature of poor self-perceived health, multilevel conceptual models are needed to explain the interaction of risk factors at different levels. This study implemented an integrated theoretical framework combining individual and contextual theories on poor health, arranging variables on logical temporal order, guiding the adjusted analysis, and, consequently, improving the estimations of the effects of locality-, family-, and individual-related characteristics of poor health self-perception. Our study showed an association between context-related variables and self-rated health status in a Colombian urban population. Further studies will be required to confirm these associations in different populations (rural, from other Colombian geographical regions or Latin American metropolitan areas), study designs, and health-related outcomes. These findings suggest that health policies and interventions aimed at improving people’s health and quality of life should include integral and multisector initiatives according to specific needs per area. Area-based strategies should take into account concerted approaches ensuring a focus on context-related variables rather than on individual-level strategies alone. Implementing these area-based strategies in Colombia might help towards the achievement of the SDGs and the Colombian Public Health Plan. To date, no study in Colombia has simultaneously considered the effect of locality, family, and individual factors on adult health perception and such approach has been scarcely used in low- and middle-income countries. Therefore, we hope our paper will contribute to the understanding of these associations in an urban area in Colombia in favour of policy-making and interventions to improve the health and well-being of individuals living in urban environments.
